# Supplementary material for: Optimal treatment options for acne scars in patients with historic acne: a systematic review and network meta-analysis
Source: PeerJ. 2025 Oct 8;13:e19938. doi: 10.7717/peerj.19938 (PMC12515001; doi:10.7717/peerj.19938)
Supplement: Supplemental Information 1 [file peerj-13-19938-s001.docx]

**VAS**

| study | treatment | mean | std.dev | sampleSize |
| --- | --- | --- | --- | --- |
| Porwal S 2018 | 9 | 6.5 | 1.56 | 27 |
| Porwal S 2018 | 1 | 4.69 | 1.59 | 28 |
| Taleb E 2024 | 2 | 5.3 | 1.21 | 16 |
| Taleb E 2024 | 5 | 5.1 | 1.53 | 16 |
| Gita Faghihi et al. 2015 | 14 | 7.8 | 1.6 | 42 |
| Gita Faghihi et al. 2015 | 2 | 6.8 | 1.9 | 42 |
| Kristoffer Hendel et al. 2023 | 2 | 4.88 | 2.7 | 15 |
| Kristoffer Hendel et al. 2023 | 1 | 6.46 | 2.86 | 15 |
| Shi‐Liu Huang et al. 2023 | 12 | 7.05 | 1.63 | 30 |
| Shi‐Liu Huang et al. 2023 | 2 | 6.95 | 1.43 | 30 |
| Rong Guo 2022 | 10 | 2.93 | 1.38 | 52 |
| Rong Guo 2022 | 2 | 1.58 | 0.92 | 57 |
| Yu jie Zhang 2022 | 12 | 3.7 | 1.13 | 20 |
| Yu jie Zhang 2022 | 2 | 3.65 | 1.08 | 20 |
| Shiwangi Rana 2017 | 1 | 6 | 0.86 | 30 |
| Shiwangi Rana 2017 | 3 | 6.79 | 1.05 | 30 |
| Zhi Yang 2019 | 14 | 7.85 | 0.26 | 20 |
| Zhi Yang 2019 | 2 | 6.85 | 0.26 | 20 |
| Amira A.M. Emam 2021 | 1 | 0.33 | 0.58 | 21 |
| Amira A.M. Emam 2021 | 2 | 9.8 | 0.51 | 21 |
| Gita Faghihi et al. 2017 | 14 | 6 | 2.2 | 25 |
| Gita Faghihi et al. 2017 | 1 | 5.1 | 1.6 | 25 |

**AE Edema**

| study | treatment | responders | sampleSize |
| --- | --- | --- | --- |
| Pooja T 2020 | 2 | 10 | 20 |
| Pooja T 2020 | 1 | 5 | 20 |
| Pooja T 2020 | 4 | 1 | 20 |
| Porwal S 2018 | 9 | 8 | 27 |
| Porwal S 2018 | 1 | 7 | 28 |
| Aya Reda Mohamed Hawwas et al. 2023 | 2 | 2 | 30 |
| Aya Reda Mohamed Hawwas et al. 2023 | 12 | 1 | 30 |
| Guo 2023 | 13 | 2 | 39 |
| Guo 2023 | 2 | 2 | 42 |
| Liao 2023 | 11 | 1 | 50 |
| Liao 2023 | 2 | 2 | 50 |
| Rong Guo et al. 2022 | 10 | 3 | 52 |
| Rong Guo et al. 2022 | 2 | 1 | 57 |
| Yu jie Zhang 2022 | 12 | 17 | 20 |
| Yu jie Zhang 2022 | 2 | 17 | 20 |
| Yuyan Wang 2021 | 13 | 25 | 350 |
| Yuyan Wang 2021 | 2 | 35 | 350 |
| Saurabh Sharma 2020 | 9 | 5 | 40 |
| Saurabh Sharma 2020 | 1 | 5 | 40 |
| Sahar A. Ismail 2022 A | 9 | 10 | 15 |
| Sahar A. Ismail 2022 A | 1 | 11 | 15 |
| Sahar A. Ismail 2022 B | 9 | 8 | 15 |
| Sahar A. Ismail 2022 B | 4 | 10 | 15 |

**AE Erythema**

| study | treatment | responders | sampleSize |
| --- | --- | --- | --- |
| Pooja T 2020 | 2 | 10 | 20 |
| Pooja T 2020 | 1 | 5 | 20 |
| Pooja T 2020 | 4 | 1 | 20 |
| Porwal S 2018 | 9 | 6 | 27 |
| Porwal S 2018 | 1 | 4 | 28 |
| Prathyusha Y 2024 | 9 | 29 | 30 |
| Prathyusha Y 2024 | 1 | 29 | 30 |
| Aya Reda Mohamed Hawwas et al. 2023 | 2 | 4 | 30 |
| Aya Reda Mohamed Hawwas et al. 2023 | 12 | 2 | 30 |
| Amr N. Saadawi et al. 2018 | 1 | 3 | 10 |
| Amr N. Saadawi et al. 2018 | 3 | 1 | 10 |
| Amr N. Saadawi et al. 2018 | 8 | 3 | 10 |
| Moustafa A. El-Taieb et al. 2019 | 4 | 3 | 25 |
| Moustafa A. El-Taieb et al. 2019 | 2 | 1 | 25 |
| Moustafa A. El-Taieb et al. 2019 | 13 | 2 | 25 |
| Ali 2019 | 1 | 4 | 20 |
| Ali 2019 | 3 | 3 | 20 |
| Ali 2019 | 8 | 2 | 20 |
| Jangir 2023 | 2 | 1 | 25 |
| Jangir 2023 | 3 | 2 | 25 |
| Yuan 2023 | 11 | 1 | 60 |
| Yuan 2023 | 2 | 6 | 60 |
| Rong Guo et al. 2022 | 10 | 3 | 52 |
| Rong Guo et al. 2022 | 2 | 3 | 57 |
| Yu jie Zhang 2022 | 12 | 19 | 20 |
| Yu jie Zhang 2022 | 2 | 19 | 20 |
| Yuyan Wang 2021 | 13 | 21 | 350 |
| Yuyan Wang 2021 | 2 | 30 | 350 |
| Shiwangi Rana 2017 | 1 | 23 | 30 |
| Shiwangi Rana 2017 | 3 | 25 | 30 |
| Saurabh Sharma 2021 | 13 | 19 | 30 |
| Saurabh Sharma 2021 | 2 | 27 | 30 |
| Amira A. Zayed 2021 | 1 | 5 | 20 |
| Amira A. Zayed 2021 | 3 | 4 | 20 |
| Zhi Yang 2019 | 14 | 4 | 20 |
| Zhi Yang 2019 | 2 | 2 | 20 |
| Saurabh Sharma 2020 | 9 | 6 | 40 |
| Saurabh Sharma 2020 | 1 | 6 | 40 |
| Sahar A. Ismail 2022 A | 9 | 10 | 15 |
| Sahar A. Ismail 2022 A | 1 | 10 | 15 |
| Sahar A. Ismail 2022 B | 9 | 11 | 15 |
| Sahar A. Ismail 2022 B | 4 | 11 | 15 |
| Chen 2024 | 11 | 10 | 12 |
| Chen 2024 | 10 | 5 | 12 |
| Chae 2014 | 2 | 5 | 20 |
| Chae 2014 | 1 | 3 | 20 |

**AE PIH**

| study | treatment | responders | sampleSize |
| --- | --- | --- | --- |
| Pooja T 2020 | 2 | 4 | 20 |
| Pooja T 2020 | 1 | 1 | 20 |
| Pooja T 2020 | 4 | 1 | 20 |
| Porwal S 2018 | 9 | 2 | 27 |
| Porwal S 2018 | 1 | 1 | 28 |
| Prathyusha Y 2024 | 9 | 2 | 30 |
| Prathyusha Y 2024 | 1 | 2 | 30 |
| Zhang Z 2013 | 1 | 1 | 33 |
| Zhang Z 2013 | 2 | 11 | 33 |
| Aya Reda Mohamed Hawwas et al. 2023 | 2 | 3 | 30 |
| Aya Reda Mohamed Hawwas et al. 2023 | 12 | 1 | 30 |
| Moustafa A. El-Taieb et al. 2019 | 4 | 5 | 25 |
| Moustafa A. El-Taieb et al. 2019 | 2 | 2 | 25 |
| Moustafa A. El-Taieb et al. 2019 | 13 | 4 | 25 |
| Ahmed 2014 | 2 | 2 | 14 |
| Ahmed 2014 | 3 | 9 | 14 |
| Bano 2023 | 1 | 1 | 30 |
| Bano 2023 | 19 | 1 | 30 |
| Cachafeiro 2016 | 1 | 1 | 20 |
| Cachafeiro 2016 | 2 | 3 | 20 |
| Yu jie Zhang 2022 | 12 | 2 | 20 |
| Yu jie Zhang 2022 | 2 | 2 | 20 |
| Yuyan Wang 2021 | 13 | 14 | 350 |
| Yuyan Wang 2021 | 2 | 35 | 350 |
| Saurabh Sharma 2021 | 13 | 6 | 30 |
| Saurabh Sharma 2021 | 2 | 10 | 30 |
| Amira A. Zayed 2021 | 1 | 4 | 20 |
| Amira A. Zayed 2021 | 3 | 2 | 20 |
| Zhi Yang 2019 | 14 | 3 | 20 |
| Zhi Yang 2019 | 2 | 2 | 20 |
| Saurabh Sharma 2020 | 9 | 1 | 40 |
| Saurabh Sharma 2020 | 1 | 1 | 40 |
| Chae 2014 | 2 | 2 | 20 |
| Chae 2014 | 1 | 1 | 20 |

**ECCA**

| study | treatment | mean | std.dev | sampleSize |
| --- | --- | --- | --- | --- |
| Zhang Z 2013 | 1 | -28.8 | 12.38870453 | 33 |
| Zhang Z 2013 | 2 | -28.9 | 13.08166656 | 33 |
| Jaishree Sharad et al. 2011 | 1 | -31.34 | 29.92815524 | 15 |
| Jaishree Sharad et al. 2011 | 8 | -62 | 29.22732887 | 15 |
| Shi‐Liu Huang et al. 2023 | 12 | -60.17 | 30.13371368 | 30 |
| Shi‐Liu Huang et al. 2023 | 2 | -45 | 28.91543014 | 30 |
| Deepika 2024 | 8 | -40.5 | 41.59 | 30 |
| Deepika 2024 | 9 | -47.8 | 37.5 | 30 |
| Yuan 2023 | 10 | -37.247 | 5.854580344 | 60 |
| Yuan 2023 | 2 | -41.891 | 7.167320908 | 60 |
| Liao 2023 | 11 | -25.397 | 3.443386269 | 50 |
| Liao 2023 | 2 | -23.171 | 3.897625944 | 50 |
| Meghna Gupta 2021 | 1 | -25.39 | 29.30939099 | 36 |
| Meghna Gupta 2021 | 4 | -23.33 | 22.77244607 | 36 |
| Yu jie Zhang 2022 | 12 | -34.75 | 19.20352311 | 20 |
| Yu jie Zhang 2022 | 2 | -24.75 | 18.3109257 | 20 |
| Yuyan Wang 2021 | 13 | -40.45 | 6.097565088 | 350 |
| Yuyan Wang 2021 | 2 | -19.91 | 6.143850584 | 350 |
| Shiwangi Rana 2017 | 1 | -29.58 | 29.69040923 | 30 |
| Shiwangi Rana 2017 | 3 | -39.65 | 30.62662241 | 30 |
| Amira A. Zayed 2021 | 1 | -14.25 | 25.2078341 | 20 |
| Amira A. Zayed 2021 | 3 | -14.45 | 20.27305601 | 20 |
| Zhi Yang 2019 | 14 | -17.27 | 14.03733949 | 20 |
| Zhi Yang 2019 | 2 | -8.17 | 12.78030907 | 20 |
| Hye Sung Han 2021 | 10 | -19.8 | 41.98361942 | 9 |
| Hye Sung Han 2021 | 2 | -5.76 | 41.23526282 | 9 |
| Sahar A. Ismail A 2022 | 9 | -38.67 | 27.31613443 | 15 |
| Sahar A. Ismail A 2022 | 1 | -42.67 | 24.37389382 | 15 |
| Sahar A. Ismail B 2022 | 9 | -10 | 26.4944296 | 15 |
| Sahar A. Ismail B 2022 | 4 | -44 | 27.30419931 | 15 |
| Hyuck Hoon KWON 2017 | 14 | -75.6 | 24.90441728 | 28 |
| Hyuck Hoon KWON 2017 | 2 | -46.2 | 24.65015213 | 28 |
| Chen 2024 | 11 | -7.09 | 12.61074542 | 12 |
| Chen 2024 | 10 | -13.34 | 12.87032634 | 12 |
| Chae 2014 | 2 | -18.75 | 24.2954399 | 20 |
| Chae 2014 | 1 | -12.75 | 25.59707014 | 20 |

**GBS**

| study | treatment | mean | std.dev | sampleSize |
| --- | --- | --- | --- | --- |
| Medhat W 2024 | 5 | -4.42 | 3.74 | 26 |
| Medhat W 2024 | 2 | -2.46 | 3.8 | 26 |
| Porwal S 2018 | 9 | -25.79 | 5.77 | 27 |
| Porwal S 2018 | 1 | -20.11 | 6.11 | 28 |
| Afra 2018 | 1 | -2 | 4.710318461 | 30 |
| Afra 2018 | 5 | -1.92 | 3.417425932 | 30 |
| Arsiwala 2020 | 13 | -6.91 | 2.994144285 | 12 |
| Arsiwala 2020 | 2 | -8.93 | 2.73 | 13 |
| Behrangi 2021 | 1 | -5.45 | 2.689981413 | 30 |
| Behrangi 2021 | 9 | -5.3 | 3.022714012 | 22 |
| Behrangi 2021 | 2 | -3.23 | 2.762173058 | 26 |
| Bano 2023 | 1 | -6.83 | 7.546263976 | 30 |
| Bano 2023 | 19 | -6.9 | 7.471351953 | 30 |
| Diab 2023 | 4 | -2.86 | 6.307305288 | 15 |
| Diab 2023 | 9 | -4.86 | 5.982608127 | 15 |
| Cachafeiro 2016 | 1 | -4.05 | 4.40658598 | 20 |
| Cachafeiro 2016 | 2 | -3.41 | 3.840130206 | 22 |
| Jangir 2023 | 2 | -6.08 | 5.880382641 | 25 |
| Jangir 2023 | 3 | -5 | 7.082224227 | 25 |
| Priya 2023 | 13 | -3.44 | 2.029014539 | 32 |
| Priya 2023 | 2 | -3.37 | 1.975018987 | 32 |
| Marwa Mohamed Kamel 2021 | 1 | -5.45 | 1.899341991 | 20 |
| Marwa Mohamed Kamel 2021 | 4 | -5.4 | 2.013082214 | 20 |
| Maryam Mumtaz 2021 | 4 | -21.92 | 4.664857983 | 46 |
| Maryam Mumtaz 2021 | 3 | -21.13 | 4.66177005 | 46 |
| Huma Saleem 2022 | 11 | -32.02 | 13.4745538 | 300 |
| Huma Saleem 2022 | 2 | -28.81 | 14.36509311 | 300 |
| Indriana Pratiwi 2020 | 10 | -7 | 8.667358306 | 18 |
| Indriana Pratiwi 2020 | 2 | -3.1 | 9.781446723 | 18 |
| V Raj Gopal Chary 2022 | 1 | -10.14 | 11.62103266 | 90 |
| V Raj Gopal Chary 2022 | 2 | -21.92 | 9.761500909 | 90 |
| Amira A.M. Emam 2021 | 1 | -6.1 | 3.65 | 21 |
| Amira A.M. Emam 2021 | 2 | -6.14 | 3.37 | 21 |

**Patient satisfaction**

| study | treatment | responders | sampleSize |
| --- | --- | --- | --- |
| A. S. Nandini 2021 | 9 | 87 | 100 |
| A. S. Nandini 2021 | 1 | 93 | 100 |
| Abdel Aal 2018 | 2 | 6 | 30 |
| Abdel Aal 2018 | 13 | 16 | 30 |
| Abdelwahab 2022 | 17 | 17 | 20 |
| Abdelwahab 2022 | 18 | 19 | 20 |
| Abdelwahab 2022 | 15 | 10 | 20 |
| Abou Eitta 2019 | 7 | 8 | 10 |
| Abou Eitta 2019 | 2 | 7 | 10 |
| Ahmed 2014 | 2 | 12 | 14 |
| Ahmed 2014 | 3 | 9 | 14 |
| Amer 2021 | 9 | 18 | 41 |
| Amer 2021 | 19 | 8 | 41 |
| Amr N. Saadawi et al. 2018 | 1 | 8 | 10 |
| Amr N. Saadawi et al. 2018 | 3 | 4 | 10 |
| Amr N. Saadawi et al. 2018 | 8 | 7 | 10 |
| Anupama 2016 | 18 | 20 | 25 |
| Anupama 2016 | 2 | 16 | 25 |
| Aya Reda Mohamed Hawwas et al. 2023 | 2 | 15 | 30 |
| Aya Reda Mohamed Hawwas et al. 2023 | 12 | 23 | 30 |
| Brar 2024 | 2 | 24 | 25 |
| Brar 2024 | 1 | 19 | 25 |
| Deepika 2024 | 8 | 19 | 30 |
| Deepika 2024 | 9 | 23 | 30 |
| Diab 2023 | 4 | 8 | 15 |
| Diab 2023 | 9 | 1 | 15 |
| Ebrahim 2021 | 15 | 21 | 34 |
| Ebrahim 2021 | 17 | 29 | 34 |
| El-Domyati M 2018 | 9 | 4 | 8 |
| El-Domyati M 2018 | 8 | 7 | 8 |
| El-Domyati M 2018 | 1 | 4 | 8 |
| Gita Faghihi et al. 2016 | 13 | 9 | 16 |
| Gita Faghihi et al. 2016 | 2 | 7 | 16 |
| Guo 2023 | 2 | 31 | 42 |
| Guo 2023 | 13 | 36 | 39 |
| Kristoffer Hendel et al. 2023 | 2 | 12 | 15 |
| Kristoffer Hendel et al. 2023 | 1 | 9 | 15 |
| Mai Abdel Raouf Osman 2016 | 2 | 22 | 30 |
| Mai Abdel Raouf Osman 2016 | 1 | 12 | 30 |
| Marwa Mohamed Kamel 2021 | 20 | 19 | 20 |
| Marwa Mohamed Kamel 2021 | 23 | 19 | 20 |
| Medhat W 2024 | 5 | 21 | 26 |
| Medhat W 2024 | 2 | 18 | 26 |
| Mehak Mukhtar et al. 2023 | 2 | 83 | 94 |
| Mehak Mukhtar et al. 2023 | 1 | 79 | 94 |
| Moustafa A. El-Taieb et al. 2019 | 4 | 7 | 25 |
| Moustafa A. El-Taieb et al. 2019 | 2 | 15 | 25 |
| Moustafa A. El-Taieb et al. 2019 | 13 | 18 | 25 |
| Priya 2023 | 13 | 27 | 32 |
| Priya 2023 | 2 | 24 | 32 |
| QURATULAIN MEMON 2022 | 1 | 17 | 40 |
| QURATULAIN MEMON 2022 | 3 | 8 | 40 |
| QURATULAIN MEMON 2022 | 8 | 33 | 40 |
| Rong Guo et al. 2022 | 10 | 48 | 52 |
| Rong Guo et al. 2022 | 2 | 45 | 57 |
| Sage 2011 | 6 | 5 | 10 |
| Sage 2011 | 15 | 8 | 10 |
| Shi‐Liu Huang et al. 2023 | 12 | 24 | 30 |
| Shi‐Liu Huang et al. 2023 | 2 | 17 | 30 |
| V Raj Gopal Chary 2022 | 1 | 81 | 90 |
| V Raj Gopal Chary 2022 | 2 | 69 | 70 |
| Vinma H. Shetty 2021 | 7 | 8 | 12 |
| Vinma H. Shetty 2021 | 20 | 8 | 12 |
| Yang 2016 | 2 | 11 | 26 |
| Yang 2016 | 5 | 6 | 26 |
| Yu jie Zhang 2022 | 12 | 10 | 20 |
| Yu jie Zhang 2022 | 2 | 2 | 20 |
| Zhang Z 2013 | 1 | 22 | 33 |
| Zhang Z 2013 | 2 | 20 | 33 |
| Zhi Yang 2019 | 14 | 18 | 20 |
| Zhi Yang 2019 | 2 | 18 | 20 |
