# Supplementary material for: Optimal treatment options for acne scars in patients with historic acne: a systematic review and network meta-analysis
Source: PeerJ. 2025 Oct 8;13:e19938. doi: 10.7717/peerj.19938 (PMC12515001; doi:10.7717/peerj.19938)
Supplement: Supplemental Information 3 — PRISMA checklist for network meta-analysis. Search strategy for network meta-analysis. Characteristics of the included studies. [file peerj-13-19938-s003.docx]

Supplementary Material

Content

[1 File S1: PRISMA checklist for network meta-analysis. 1](#_Toc8681)

[2 File S2: Search strategy for network meta-analysis. 5](#_Toc30404)

[2.1 Search strategy of Pubmed. 5](#_Toc10105)

[2.2 Search strategy of Embase. 8](#_Toc26807)

[2.3 Search strategy of Cochrane Library. 11](#_Toc18266)

[2.4 Search strategy of Web of Science. 14](#_Toc12047)

[3 File S3: Characteristics of the included studies. 16](#_Toc27907)

[4 Target Audience Description 28](#_Toc5151)

# File S1: PRISMA checklist for network meta-analysis.

| **Section/topic** | **#** | **Checklist item** | **Reported on page #** |
| --- | --- | --- | --- |
| **TITLE** | | |  |
| Title | 1 | Identify the report as a systematic review incorporating a network meta-analysis (or related form of  meta-analysis). | Page 1 |
| **ABSTRACT** | | |  |
| Structured summary | 2 | Provide a structured summary including, as applicable:  Background: main objectives  Methods: data sources; study eligibility criteria, participants, and interventions; study appraisal; and synthesis methods, such as network meta-analysis.  Results: number of studies and participants identified; summary estimates with corresponding confidence/credible intervals; treatment rankings may also be discussed. Authors may choose to summarize pairwise comparisons against a chosen treatment included in their analyses for brevity.  Discussion/Conclusions: limitations; conclusions and implications of findings.  Other: primary source of funding; systematic review registration number with registry name. | Page 1 |
| **INTRODUCTION** | | |  |
| Rationale | 3 | Describe the rationale for the review in the context of what is already known, including mention of why a network meta-analysis has been conducted | Page 2 |
| Objectives | 4 | Provide an explicit statement of questions being addressed with reference to participants, interventions, comparisons, outcomes, and study design (PICOS). | Page 2 |
| **METHODS** | | |  |
| Protocol and registration | 5 | Indicate if a review protocol exists and if and where it can be accessed (e.g., Web address), and, if available, provide registration information including registration number. | Page 3 |
| Eligibility criteria | 6 | Specify study characteristics (e.g., PICOS, length of follow-up) and report characteristics (e.g., years considered, language, publication status) used as criteria for eligibility, giving rationale. Clearly describe eligible treatments included in the treatment network and note whether any have been clustered or merged into the same node (with justification). | Page 3 Section 2 |
| Information sources | 7 | Describe all information sources (e.g., databases with dates of coverage, contact with study authors to identify additional studies) in the search and date last searched. | Page 3 Section 1 |
| Search | 8 | Present full electronic search strategy for at least one database, including any limits used, such that it could be repeated. | Supplementary File S2 |
| Study selection | 9 | State the process for selecting studies (i.e., screening, eligibility, included in systematic review, and, if applicable, included in the meta-analysis). | Page 3 Section 3 |
| Data collection process | 10 | Describe method of data extraction from reports (e.g., piloted forms, independently, in duplicate) and any processes for obtaining and confirming data from investigators. | Page 3 Section 3 |
| Data items | 11 | List and define all variables for which data were sought (e.g., PICOS, funding sources) and any assumptions and simplifications made. | Page 3 Section 2 |
| Geometry of the network | S1 | Describe methods used to explore the geometry of the treatment network under study and potential biases related to it. This should include how the evidence base has been graphically summarized for presentation, and what characteristics were compiled and used to describe the evidence base to readers | Page 4 Section 5 |
| Risk of bias within individual studies | 12 | Describe methods used for assessing risk of bias of individual studies (including specification of whether this was done at the study or outcome level), and how this information is to be used in any data synthesis. | Page 3-4 Section 4 |
| Summary measures | 13 | State the principal summary measures (e.g., risk ratio, difference in means). Also describe the use of additional summary measures assessed, such as treatment rankings and surface under the cumulative ranking curve (SUCRA) values, as well as modified approaches used to present summary findings from meta-analyses. | Page 4 Section 5 |
| Planned methods of analysis | 14 | Describe the methods of handling data and combining results of studies for each network meta-analysis. This should include, but not be limited to: Handling of multigroup trials; Selection of variance structure; Selection of prior distributions in Bayesian analyses; and Assessment of model fit. | Page 4 Section 5 |
| Assessment of inconsistency | S2 | Describe the statistical methods used to evaluate the agreement of direct and indirect evidence in the treatment network(s) studied. Describe efforts taken to address its presence when found. | Page 4 Section 5 |
| Risk of bias across studies | 15 | Specify any assessment of risk of bias that may affect the cumulative evidence (e.g., publication bias, selective reporting within studies) | Page 4 Section 5 |
| Additional analyses | 16 | Describe methods of additional analyses if done, indicating which were prespecified. This may include, but not be limited to, the following: Sensitivity or subgroup analyses; Meta-regression analyses; Alternative formulations of the treatment network; and Use of alternative prior distributions for Bayesian analyses (if applicable). | Page 4 Section 5 |
| **RESULTS** | | |  |
| Study selection | 17 | Give numbers of studies screened, assessed for eligibility, and included in the review, with reasons for exclusions at each stage, ideally with a flow diagram. | Page 4-5 Section 1 |
| Presentation of network structure | S3 | Provide a network graph of the included studies to enable visualization of the geometry of the treatment network. | Figure 3-4 |
| Summary of network geometry | S4 | Provide a brief overview of characteristics of the treatment network. This may include commentary on the abundance of trials and randomized patients for the different interventions and pairwise comparisons in the network, gaps of evidence in the treatment network, and potential biases reflected by the network structure. | Page 5 Section 2 |
| Study characteristics | 18 | For each study, present characteristics for which data were extracted (e.g., study size, PICOS, follow-up period) and provide the citations. | Supplementary File S2 |
| Risk of bias within studies | 19 | Present data on risk of bias of each study and, if available, any outcome level assessment. | Page 5 Section 2 |
| Results of individual studies | 20 | For all outcomes considered (benefits or harms), present, for each study: 1) simple summary data for each intervention group, and 2) effect estimates and confidence intervals. Modified approaches may be needed to deal with information from larger networks. | Page 5 Section 1-2 |
| Synthesis of results | 21 | Present results of each meta-analysis done, including confidence/credible intervals. In larger networks, authors may focus on comparisons versus a particular comparator (e.g., placebo or standard care), with full findings presented in an appendix. League tables and forest plots may be considered to summarize pairwise comparisons. If additional summary measures were explored (such as treatment rankings), these should also be presented. | Page 5-7 Section 3.1-3.6.3 |
| Exploration for inconsistency | S5 | Describe results from investigations of inconsistency. This may include such information as measures of model fit to compare consistency and inconsistency models, P values from statistical tests, or summary of inconsistency estimates from different parts of the treatment network. | Table 1 |
| Risk of bias across studies | 22 | Present results of any assessment of risk of bias across studies for the evidence base being studied. | Page 7 Section 3.7 |
| Results of additional analyses | 23 | Give results of additional analyses, if done (e.g., sensitivity or subgroup analyses, meta-regression  analyses, alternative network geometries studied, alternative choice of prior distributions for  Bayesian analyses, and so forth). | Page 5 Section 2 |
| **DISCUSSION** | | |  |
| Summary of evidence | 24 | Summarize the main findings, including the strength of evidence for each main outcome; consider their relevance to key groups (e.g., health care providers, researchers, and policymakers). | Page 7-9 |
| Limitations | 25 | Discuss limitations at study and outcome level (e.g., risk of bias), and at review level (e.g., incomplete retrieval of identified research, reporting bias). Comment on the validity of the assumptions, such as transitivity and consistency. Comment on any concerns regarding network geometry (e.g., avoidance of certain comparisons). | Page 9 |
| Conclusions | 26 | Provide a general interpretation of the results in the context of other evidence, and implications for future research. | Page 10 |
| **FUNDING** | | |  |
| Funding | 27 | Describe sources of funding for the systematic review and other support (e.g., supply of data); role of funders for the systematic review. This should also include information regarding whether funding has been received from manufacturers of treatments in the network and/or whether some of the authors are content experts with professional conflicts of interest that could affect use of treatments in the network. | Page 10 |

# File S2: Search strategy for network meta-analysis.

## Search strategy of Pubmed.

| No. | Search items |
| --- | --- |
| #1 | (((((Lasers, Solid-State[MeSH Terms]) OR (Lasers, Gas[MeSH Terms])) OR (Radiofrequency Therapy[MeSH Terms])) OR (Laser Therapy[MeSH Terms])) OR (Platelet-Rich Plasma[MeSH Terms])) OR (Transplantation[MeSH Terms]) |
| #2 | "Acupulse"[Title/Abstract] OR "Affirm neodymium YAG laser"[Title/Abstract] OR "Alexandrite Laser*"[Title/Abstract] OR "Argon Ion Laser*"[Title/Abstract] OR "Autologous Fat Grafting"[Title/Abstract] OR "Autologous Fat Transfer"[Title/Abstract] OR "Carbon Dioxide Laser"[Title/Abstract] OR "carbon dioxide laser device"[Title/Abstract] OR "carbon dioxide laser systems"[Title/Abstract] OR "Carbon Dioxide Lasers"[Title/Abstract] OR "Chemotherap*"[Title/Abstract] OR "CO2 Laser"[Title/Abstract] OR "CO2 Lasers"[Title/Abstract] OR "continuous wave carbon dioxide laser"[Title/Abstract] OR "Copper Vapor Laser*"[Title/Abstract] OR "CT3 Plus"[Title/Abstract] OR "Diode Pumped Solid State Laser*"[Title/Abstract] OR "dLase 300"[Title/Abstract] OR "Drug Therap*"[Title/Abstract] OR "drug treatment"[Title/Abstract] OR "Ds-40u"[Title/Abstract] OR "eCO2"[Title/Abstract] OR "Er YAG Laser*"[Title/Abstract] OR "Erbium YAG Laser*"[Title/Abstract] OR "Erbium Doped Yttrium Aluminum Garnet Laser*"[Title/Abstract] OR "Er-YAG Laser*"[Title/Abstract] OR "Fractional Micro-needle Radiofrequency"[Title/Abstract] OR "Fractional Micro-plasma Radio Frequency"[Title/Abstract] OR "Fraxel Re:fine"[Title/Abstract] OR "Gas Laser"[Title/Abstract] OR "Gas Lasers"[Title/Abstract] OR "general use solid state carbon dioxide laser"[Title/Abstract] OR "general/multiple surgical solid-state/carbon dioxide laser system"[Title/Abstract] OR "Ghost Surgery"[Title/Abstract] OR "Gold Vapor Laser*"[Title/Abstract] OR "graft surgery"[Title/Abstract] OR "grafting surgery"[Title/Abstract] OR "Helium Laser*"[Title/Abstract] OR "Helium Neon Gas Lasers"[Title/Abstract] OR "HeNe Laser*"[Title/Abstract] OR "Ho YAG Laser*"[Title/Abstract] OR "Holmium Laser*"[Title/Abstract] OR "Holmium YAG Laser*"[Title/Abstract] OR "Holmium Doped Yttrium Aluminum Garnet Lasers"[Title/Abstract] OR "Holmium-YAG Laser*"[Title/Abstract] OR "Ho-YAG Laser*"[Title/Abstract] OR "JOULE ClearSense"[Title/Abstract] OR "Juvia"[Title/Abstract] OR "KTP Laser*"[Title/Abstract] OR "LASER therapy"[Title/Abstract] OR "laser treatment"[Title/Abstract] OR "Laser Ablation"[Title/Abstract] OR "Laser Knife*"[Title/Abstract] OR "Laser Knive*"[Title/Abstract] OR "Laser Photoablation of Tissue"[Title/Abstract] OR "Laser Scalpel*"[Title/Abstract] OR "Laser Surger*"[Title/Abstract] OR "Laser Therap*"[Title/Abstract] OR "Laser Tissue Ablation"[Title/Abstract] OR "Laser Vaporization"[Title/Abstract] OR "Lasertronics Paragon 50"[Title/Abstract] OR "light amplification by stimulated emission of radiation therapy "[Title/Abstract] OR "medicament therapy"[Title/Abstract] OR "medicament treatment"[Title/Abstract] OR "medication"[Title/Abstract] OR "medicinal therapy"[Title/Abstract] OR "medicinal treatment"[Title/Abstract] OR "Metal Vapor Laser*"[Title/Abstract] OR "Mosaic CO2"[Title/Abstract] OR "Nd YAG Laser*"[Title/Abstract] OR "Nd:YAG laser"[Title/Abstract] OR "Nd-YAG Laser*"[Title/Abstract] OR "neodymium doped yttrium aluminum garnet laser"[Title/Abstract] OR "neodymium YAG laser"[Title/Abstract] OR "neodymium yttrium aluminium garnet laser"[Title/Abstract] OR "neodymium yttrium aluminum garnet laser"[Title/Abstract] OR "Neodymium Doped Yttrium Aluminum Garnet Laser*"[Title/Abstract] OR "neodymium:YAG laser"[Title/Abstract] OR "Neodymium-Doped Yttrium Aluminum Garnet Laser*"[Title/Abstract] OR "Nitrogen Laser"[Title/Abstract] OR "Nitrogen Lasers"[Title/Abstract] OR "Non-ablative Fractional Erbium Glass Laser"[Title/Abstract] OR "Nonablative Laser Treatment"[Title/Abstract] OR "Nonablative Laser Treatments"[Title/Abstract] OR "Opelaser-03 S"[Title/Abstract] OR "operation"[Title/Abstract] OR "operation care"[Title/Abstract] OR "operative intervention"[Title/Abstract] OR "Operative Procedure"[Title/Abstract] OR "Operative Procedures"[Title/Abstract] OR "operative repair"[Title/Abstract] OR "operative restoration"[Title/Abstract] OR "operative surgery"[Title/Abstract] OR "Operative Surgical Procedure"[Title/Abstract] OR "operative treatment"[Title/Abstract] OR "Periolase MVP7"[Title/Abstract] OR "pharmaceutical therapy"[Title/Abstract] OR "pharmaceutical treatment"[Title/Abstract] OR "pharmacological therapy"[Title/Abstract] OR "pharmacological treatment"[Title/Abstract] OR "Pharmacotherapies"[Title/Abstract] OR "Pharmacotherapy"[Title/Abstract] OR "pharmaco-therapy"[Title/Abstract] OR "pharmacotreatment"[Title/Abstract] OR "pharmaco-treatment"[Title/Abstract] OR "PinPointe FootLaser"[Title/Abstract] OR "Pixel Perfect"[Title/Abstract] OR "Platelet Rich Plasma"[Title/Abstract] OR "Potassium Titanyl Phosphate Laser"[Title/Abstract] OR "Potassium Titanyl Phosphate Lasers"[Title/Abstract] OR "Pulsed Laser Tissue Ablation"[Title/Abstract] OR "Pulsemaster 600 IQ"[Title/Abstract] OR "Q-Clear"[Title/Abstract] OR "Radio Frequency Therapy"[Title/Abstract] OR "Radio-Frequency Therapies"[Title/Abstract] OR "radiofrequency therapy"[Title/Abstract] OR "radiofrequency treatment"[Title/Abstract] OR "radio-frequency treatment"[Title/Abstract] OR "Radiofrequency Therapies"[Title/Abstract] OR "Radiofrequency Therapy"[Title/Abstract] OR "research surgery"[Title/Abstract] OR "resection"[Title/Abstract] OR "Ruby Laser"[Title/Abstract] OR "Ruby Lasers"[Title/Abstract] OR "SCx10 Carbon Dioxide Slab Laser"[Title/Abstract] OR "serial transplantation"[Title/Abstract] OR "Smartxide"[Title/Abstract] OR "SmartXide DOT"[Title/Abstract] OR "solid state carbon dioxide laser system"[Title/Abstract] OR "Solid State Laser"[Title/Abstract] OR "Solid-State Laser"[Title/Abstract] OR "Solid-State Lasers"[Title/Abstract] OR "specialties, surgical"[Title/Abstract] OR "Spectra"[Title/Abstract] OR "Subcutaneous Excision "[Title/Abstract] OR "surgery"[Title/Abstract] OR "surgical care"[Title/Abstract] OR "surgical correction"[Title/Abstract] OR "surgical diagnosis"[Title/Abstract] OR "surgical diagnostic techniques"[Title/Abstract] OR "surgical exposure"[Title/Abstract] OR "surgical grafting"[Title/Abstract] OR "surgical intervention"[Title/Abstract] OR "surgical management"[Title/Abstract] OR "surgical operation"[Title/Abstract] OR "surgical practice"[Title/Abstract] OR "Surgical Procedure"[Title/Abstract] OR "Surgical Procedures"[Title/Abstract] OR "surgical repair"[Title/Abstract] OR "surgical research"[Title/Abstract] OR "surgical restoration"[Title/Abstract] OR "surgical service"[Title/Abstract] OR "surgical special*"[Title/Abstract] OR "surgical therapy"[Title/Abstract] OR "surgical treatment"[Title/Abstract] OR "therapeutic uses"[Title/Abstract] OR "thrombocyte rich plasma"[Title/Abstract] OR "tissue and organ procurement"[Title/Abstract] OR "transplantation surgery"[Title/Abstract] OR "Transplantation*"[Title/Abstract] OR "transplants"[Title/Abstract] OR "UltraPulse Encore"[Title/Abstract] OR "UltraPulse Surgitouch"[Title/Abstract] OR "UM-L30"[Title/Abstract] OR "Xenon Ion Laser"[Title/Abstract] OR "Xenon Ion Lasers"[Title/Abstract] OR "YAG Laser"[Title/Abstract] OR "YAG Lasers"[Title/Abstract] OR "YLF Laser"[Title/Abstract] OR "YLF Lasers"[Title/Abstract] OR "YSGG Laser"[Title/Abstract] OR "YSGG Lasers"[Title/Abstract] OR "Yttrium Aluminum Garnet Laser*"[Title/Abstract] OR "Yttrium Lithium Fluoride Laser*"[Title/Abstract] OR "Yttrium Scandium Gallium Garnet Laser*"[Title/Abstract] OR "Yttrium-Lithium-Fluoride Laser*"[Title/Abstract] OR "Yttrium-Scandium-Gallium Garnet Laser*"[Title/Abstract] |
| #3 | (Acne Vulgaris[MeSH Terms]) OR (Acne Keloid[MeSH Terms]) |
| #4 | "Acne"[Title/Abstract] OR "acne keloid*"[Title/Abstract] OR "Acne Vulgaris"[Title/Abstract] OR "acneiform eruption"[Title/Abstract] OR "acneiform eruptions"[Title/Abstract] OR "acneiform skin eruption"[Title/Abstract] OR "dermatitis papillaris capilliti*"[Title/Abstract] OR "Folliculitis Keloidalis"[Title/Abstract] OR "Folliculitis Keloidalis Nuchae"[Title/Abstract] OR "keloid acne"[Title/Abstract] OR "keloid folliculitis"[Title/Abstract] OR "keloidal acne"[Title/Abstract] OR "keloidal folliculitis"[Title/Abstract] OR "Keloidal Acne*"[Title/Abstract] OR "keloidalis nuchae acne"[Title/Abstract] OR "Lichen Keloidalis Nuchae"[Title/Abstract] OR "nuchal acne keloidalis"[Title/Abstract] OR "nuchal keloid acne"[Title/Abstract] OR "Nuchal Keloid Acnes"[Title/Abstract] OR "Nuchal Keloid Acne"[Title/Abstract] OR "pomade acne"[Title/Abstract] OR "tropical acne"[Title/Abstract] |
| #5 | Controlled Clinical Trial [Publication Type] OR Randomized Controlled Trial[Publication Type] OR Equivalence Trial[Publication Type] OR Pragmatic Clinical Trial[Publication Type] OR random*[All Fields] |
| #6 | (#1 OR #2) AND (#3 OR #4) |

## Search strategy of Embase.

| No. | Search items |
| --- | --- |
| #1 | 'acne'/exp |
| #2 | 'acne keloidalis'/exp |
| #3 | 'acne':ti,ab,kw OR 'acne keloid*':ti,ab,kw OR 'acne vulgaris':ti,ab,kw OR 'acneiform eruption':ti,ab,kw OR 'acneiform eruptions':ti,ab,kw OR 'acneiform skin eruption':ti,ab,kw OR 'dermatitis papillaris capilliti*':ti,ab,kw OR 'folliculitis keloidalis':ti,ab,kw OR 'folliculitis keloidalis nuchae':ti,ab,kw OR 'keloid acne':ti,ab,kw OR 'keloid folliculitis':ti,ab,kw OR 'keloidal acne':ti,ab,kw OR 'keloidal folliculitis':ti,ab,kw OR 'keloidal acne*':ti,ab,kw OR 'keloidalis nuchae acne':ti,ab,kw OR 'lichen keloidalis nuchae':ti,ab,kw OR 'nuchal acne keloidalis':ti,ab,kw OR 'nuchal keloid acnes':ti,ab,kw OR 'nuchal keloid acne':ti,ab,kw OR 'pomade acne':ti,ab,kw OR 'tropical acne':ti,ab,kw OR 'acne scar*':ti,ab,kw |
| #4 | 'acupulse':ti,ab,kw OR 'affirm neodymium yag laser':ti,ab,kw OR 'alexandrite laser*':ti,ab,kw OR 'argon ion laser*':ti,ab,kw OR 'autologous fat grafting':ti,ab,kw OR 'autologous fat transfer':ti,ab,kw OR 'carbon dioxide laser':ti,ab,kw OR 'carbon dioxide laser device':ti,ab,kw OR 'carbon dioxide laser systems':ti,ab,kw OR 'carbon dioxide lasers':ti,ab,kw OR 'chemotherap*':ti,ab,kw OR 'co2 laser':ti,ab,kw OR 'co2 lasers':ti,ab,kw OR 'continuous wave carbon dioxide laser':ti,ab,kw OR 'copper vapor laser*':ti,ab,kw OR 'ct3 plus':ti,ab,kw OR 'diode pumped solid state laser*':ti,ab,kw OR 'dlase 300':ti,ab,kw OR 'drug therap*':ti,ab,kw OR 'drug treatment':ti,ab,kw OR 'ds-40u':ti,ab,kw OR 'eco2':ti,ab,kw OR 'er yag laser*':ti,ab,kw OR 'erbium yag laser*':ti,ab,kw OR 'erbium doped yttrium aluminum garnet laser*':ti,ab,kw OR 'er-yag laser*':ti,ab,kw OR 'fractional micro-needle radiofrequency':ti,ab,kw OR 'fractional micro-plasma radio frequency':ti,ab,kw OR 'fraxel re:fine':ti,ab,kw OR 'gas laser':ti,ab,kw OR 'gas lasers':ti,ab,kw OR 'general use solid state carbon dioxide laser':ti,ab,kw OR 'general/multiple surgical solid-state/carbon dioxide laser system':ti,ab,kw OR 'ghost surgery':ti,ab,kw OR 'gold vapor laser*':ti,ab,kw OR 'graft surgery':ti,ab,kw OR 'grafting surgery':ti,ab,kw OR 'helium laser*':ti,ab,kw OR 'helium neon gas lasers':ti,ab,kw OR 'hene laser*':ti,ab,kw OR 'ho yag laser*':ti,ab,kw OR 'holmium laser*':ti,ab,kw OR 'holmium yag laser*':ti,ab,kw OR 'holmium doped yttrium aluminum garnet lasers':ti,ab,kw OR 'holmium-yag laser*':ti,ab,kw OR 'ho-yag laser*':ti,ab,kw OR 'joule clearsense':ti,ab,kw OR 'juvia':ti,ab,kw OR 'ktp laser*':ti,ab,kw OR 'laser therapy':ti,ab,kw OR 'laser treatment':ti,ab,kw OR 'laser ablation':ti,ab,kw OR 'laser knife*':ti,ab,kw OR 'laser knive*':ti,ab,kw OR 'laser photoablation of tissue':ti,ab,kw OR 'laser scalpel*':ti,ab,kw OR 'laser surger*':ti,ab,kw OR 'laser therap*':ti,ab,kw OR 'laser tissue ablation':ti,ab,kw OR 'laser vaporization':ti,ab,kw OR 'lasertronics paragon 50':ti,ab,kw OR 'light amplification by stimulated emission of radiation therapy':ti,ab,kw OR 'medicament therapy':ti,ab,kw OR 'medicament treatment':ti,ab,kw OR 'medication':ti,ab,kw OR 'medicinal therapy':ti,ab,kw OR 'medicinal treatment':ti,ab,kw OR 'metal vapor laser*':ti,ab,kw OR 'mosaic co2':ti,ab,kw OR 'nd yag laser*':ti,ab,kw OR 'nd:yag laser':ti,ab,kw OR 'nd-yag laser*':ti,ab,kw OR 'neodymium doped yttrium aluminum garnet laser':ti,ab,kw OR 'neodymium yag laser':ti,ab,kw OR 'neodymium yttrium aluminium garnet laser':ti,ab,kw OR 'neodymium yttrium aluminum garnet laser':ti,ab,kw OR 'neodymium doped yttrium aluminum garnet laser*':ti,ab,kw OR 'neodymium:yag laser':ti,ab,kw OR 'neodymium-doped yttrium aluminum garnet laser*':ti,ab,kw OR 'nitrogen laser':ti,ab,kw OR 'nitrogen lasers':ti,ab,kw OR 'non-ablative fractional erbium glass laser':ti,ab,kw OR 'nonablative laser treatment':ti,ab,kw OR 'nonablative laser treatments':ti,ab,kw OR 'opelaser-03 s':ti,ab,kw OR 'operation':ti,ab,kw OR 'operation care':ti,ab,kw OR 'operative intervention':ti,ab,kw OR 'operative procedure':ti,ab,kw OR 'operative procedures':ti,ab,kw OR 'operative repair':ti,ab,kw OR 'operative restoration':ti,ab,kw OR 'operative surgery':ti,ab,kw OR 'operative surgical procedure':ti,ab,kw OR 'operative treatment':ti,ab,kw OR 'periolase mvp7':ti,ab,kw OR 'pharmaceutical therapy':ti,ab,kw OR 'pharmaceutical treatment':ti,ab,kw OR 'pharmacological therapy':ti,ab,kw OR 'pharmacological treatment':ti,ab,kw OR 'pharmacotherapies':ti,ab,kw OR 'pharmacotherapy':ti,ab,kw OR 'pharmaco-therapy':ti,ab,kw OR 'pharmacotreatment':ti,ab,kw OR 'pharmaco-treatment':ti,ab,kw OR 'pinpointe footlaser':ti,ab,kw OR 'pixel perfect':ti,ab,kw OR 'platelet rich plasma':ti,ab,kw OR 'potassium titanyl phosphate laser':ti,ab,kw OR 'potassium titanyl phosphate lasers':ti,ab,kw OR 'pulsed laser tissue ablation':ti,ab,kw OR 'pulsemaster 600 iq':ti,ab,kw OR 'q-clear':ti,ab,kw OR 'radio frequency therapy':ti,ab,kw OR 'radio-frequency therapies':ti,ab,kw OR 'radiofrequency treatment':ti,ab,kw OR 'radio-frequency treatment':ti,ab,kw OR 'radiofrequency therapies':ti,ab,kw OR 'radiofrequency therapy':ti,ab,kw OR 'research surgery':ti,ab,kw OR 'resection':ti,ab,kw OR 'ruby laser':ti,ab,kw OR 'ruby lasers':ti,ab,kw OR 'scx10 carbon dioxide slab laser':ti,ab,kw OR 'serial transplantation':ti,ab,kw OR 'smartxide':ti,ab,kw OR 'smartxide dot':ti,ab,kw OR 'solid state carbon dioxide laser system':ti,ab,kw OR 'solid state laser':ti,ab,kw OR 'solid-state laser':ti,ab,kw OR 'solid-state lasers':ti,ab,kw OR 'specialties, surgical':ti,ab,kw OR 'spectra':ti,ab,kw OR 'subcutaneous excision':ti,ab,kw OR 'surgery':ti,ab,kw OR 'surgical care':ti,ab,kw OR 'surgical correction':ti,ab,kw OR 'surgical diagnosis':ti,ab,kw OR 'surgical diagnostic techniques':ti,ab,kw OR 'surgical exposure':ti,ab,kw OR 'surgical grafting':ti,ab,kw OR 'surgical intervention':ti,ab,kw OR 'surgical management':ti,ab,kw OR 'surgical operation':ti,ab,kw OR 'surgical practice':ti,ab,kw OR 'surgical procedure':ti,ab,kw OR 'surgical procedures':ti,ab,kw OR 'surgical repair':ti,ab,kw OR 'surgical research':ti,ab,kw OR 'surgical restoration':ti,ab,kw OR 'surgical service':ti,ab,kw OR 'surgical special*':ti,ab,kw OR 'surgical therapy':ti,ab,kw OR 'surgical treatment':ti,ab,kw OR 'therapeutic uses':ti,ab,kw OR 'thrombocyte rich plasma':ti,ab,kw OR 'tissue and organ procurement':ti,ab,kw OR 'transplantation surgery':ti,ab,kw OR 'transplantation*':ti,ab,kw OR 'transplants':ti,ab,kw OR 'ultrapulse encore':ti,ab,kw OR 'ultrapulse surgitouch':ti,ab,kw OR 'um-l30':ti,ab,kw OR 'xenon ion laser':ti,ab,kw OR 'xenon ion lasers':ti,ab,kw OR 'yag laser':ti,ab,kw OR 'yag lasers':ti,ab,kw OR 'ylf laser':ti,ab,kw OR 'ylf lasers':ti,ab,kw OR 'ysgg laser':ti,ab,kw OR 'ysgg lasers':ti,ab,kw OR 'yttrium aluminum garnet laser*':ti,ab,kw OR 'yttrium lithium fluoride laser*':ti,ab,kw OR 'yttrium scandium gallium garnet laser*':ti,ab,kw OR 'yttrium-lithium-fluoride laser*':ti,ab,kw OR 'yttrium-scandium-gallium garnet laser*':ti,ab,kw |
| #5 | 'neodymium yag laser'/exp OR 'carbon dioxide laser'/exp OR 'radiofrequency therapy'/exp OR 'laser therapy'/exp OR 'thrombocyte rich plasma'/exp OR 'transplantation'/exp OR 'drug therapy'/exp OR 'surgery'/exp |
| #6 | (#1 OR #2 OR #3) AND (#4 OR #5) |

## Search strategy of Cochrane Library.

| No. | Search items |
| --- | --- |
| #1 | MeSH descriptor: [Acne Keloid] explode all trees |
| #2 | MeSH descriptor: [Acne Vulgaris] explode all trees |
| #3 | (‘Acne’ OR ‘acne keloid*’ OR ‘Acne Vulgaris’ OR ‘acneiform eruption’ OR ‘acneiform eruptions’ OR ‘acneiform skin eruption’ OR ‘dermatitis papillaris capilliti*’ OR ‘Folliculitis Keloidalis’ OR ‘Folliculitis Keloidalis Nuchae’ OR ‘keloid acne’ OR ‘keloid folliculitis’ OR ‘keloidal acne’ OR ‘keloidal folliculitis’ OR ‘Keloidal Acne*’ OR ‘keloidalis nuchae acne’ OR ‘Lichen Keloidalis Nuchae’ OR ‘nuchal acne keloidalis’ OR ‘nuchal keloid acne’ OR ‘Nuchal Keloid Acnes’ OR ‘Nuchal Keloid Acne’ OR ‘pomade acne’ OR ‘tropical acne’):ti,ab,kw |
| #4 | MeSH descriptor: [Lasers, Solid-State] explode all trees |
| #5 | MeSH descriptor: [Lasers, Gas] explode all trees |
| #6 | MeSH descriptor: [Radiofrequency Therapy] explode all trees |
| #7 | MeSH descriptor: [Laser Therapy] explode all trees |
| #8 | MeSH descriptor: [Platelet-Rich Plasma] explode all trees |
| #9 | MeSH descriptor: [Transplantation] explode all trees |
| #10 | MeSH descriptor: [Drug Therapy] explode all trees |
| #11 | (‘Acupulse’ OR ‘Affirm neodymium YAG laser’ OR ‘Alexandrite Laser*’ OR ‘Argon Ion Laser*’ OR ‘Autologous Fat Grafting’ OR ‘Autologous Fat Transfer’ OR ‘Carbon Dioxide Laser’ OR ‘carbon dioxide laser device’ OR ‘carbon dioxide laser systems’ OR ‘Carbon Dioxide Lasers’ OR ‘Chemotherap*’ OR ‘CO2 Laser’ OR ‘CO2 Lasers’ OR ‘continuous wave carbon dioxide laser’ OR ‘Copper Vapor Laser*’ OR ‘CT3 Plus’ OR ‘Diode Pumped Solid State Laser*’ OR ‘dLase 300’ OR ‘Drug Therap*’ OR ‘drug treatment’ OR ‘Ds 40u’ OR ‘eCO2’ OR ‘Er YAG Laser*’ OR ‘Erbium YAG Laser*’ OR ‘Erbium Doped Yttrium Aluminum Garnet Laser*’ OR ‘Er YAG Laser*’ OR ‘Fractional Micro needle Radiofrequency’ OR ‘Fractional Micro plasma Radio Frequency’ OR ‘Fraxel Re fine’ OR ‘Gas Laser’ OR ‘Gas Lasers’ OR ‘general use solid state carbon dioxide laser’ OR ‘general multiple surgical solid state carbon dioxide laser system’ OR ‘Ghost Surgery’ OR ‘Gold Vapor Laser*’ OR ‘graft surgery’ OR ‘grafting surgery’ OR ‘Helium Laser*’ OR ‘Helium Neon Gas Lasers’ OR ‘HeNe Laser*’ OR ‘Ho YAG Laser*’ OR ‘Holmium Laser*’ OR ‘Holmium YAG Laser*’ OR ‘Holmium Doped Yttrium Aluminum Garnet Lasers’ OR ‘Holmium YAG Laser*’ OR ‘Ho YAG Laser*’ OR ‘JOULE ClearSense’ OR ‘Juvia’ OR ‘KTP Laser*’ OR ‘LASER therapy’ OR ‘laser treatment’ OR ‘Laser Ablation’ OR ‘Laser Knife*’ OR ‘Laser Knive*’ OR ‘Laser Photoablation of Tissue’ OR ‘Laser Scalpel*’ OR ‘Laser Surger*’ OR ‘Laser Therap*’ OR ‘Laser Tissue Ablation’ OR ‘Laser Vaporization’ OR ‘Lasertronics Paragon 50’ OR ‘light amplification by stimulated emission of radiation therapy ‘ OR ‘medicament therapy’ OR ‘medicament treatment’ OR ‘medication’ OR ‘medicinal therapy’ OR ‘medicinal treatment’ OR ‘Metal Vapor Laser*’ OR ‘Mosaic CO2’ OR ‘Nd YAG Laser*’ OR ‘Nd YAG laser’ OR ‘Nd YAG Laser*’ OR ‘neodymium doped yttrium aluminum garnet laser’ OR ‘neodymium YAG laser’ OR ‘neodymium yttrium aluminium garnet laser’ OR ‘neodymium yttrium aluminum garnet laser’ OR ‘Neodymium Doped Yttrium Aluminum Garnet Laser*’ OR ‘neodymium YAG laser’ OR ‘Neodymium Doped Yttrium Aluminum Garnet Laser*’ OR ‘Nitrogen Laser’ OR ‘Nitrogen Lasers’ OR ‘Non ablative Fractional Erbium Glass Laser’ OR ‘Nonablative Laser Treatment’ OR ‘Nonablative Laser Treatments’ OR ‘Opelaser 03 S’ OR ‘operation’ OR ‘operation care’ OR ‘operative intervention’ OR ‘Operative Procedure’ OR ‘Operative Procedures’ OR ‘operative repair’ OR ‘operative restoration’ OR ‘operative surgery’ OR ‘Operative Surgical Procedure’ OR ‘operative treatment’ OR ‘Periolase MVP7’ OR ‘pharmaceutical therapy’ OR ‘pharmaceutical treatment’ OR ‘pharmacological therapy’ OR ‘pharmacological treatment’ OR ‘Pharmacotherapies’ OR ‘Pharmacotherapy’ OR ‘pharmaco therapy’ OR ‘pharmacotreatment’ OR ‘pharmaco treatment’ OR ‘PinPointe FootLaser’ OR ‘Pixel Perfect’ OR ‘Platelet Rich Plasma’ OR ‘Potassium Titanyl Phosphate Laser’ OR ‘Potassium Titanyl Phosphate Lasers’ OR ‘Pulsed Laser Tissue Ablation’ OR ‘Pulsemaster 600 IQ’ OR ‘Q Clear’ OR ‘Radio Frequency Therapy’ OR ‘Radio Frequency Therapies’ OR ‘radiofrequency therapy’ OR ‘radiofrequency treatment’ OR ‘radio frequency treatment’ OR ‘Radiofrequency Therapies’ OR ‘Radiofrequency Therapy’ OR ‘research surgery’ OR ‘resection’ OR ‘Ruby Laser’ OR ‘Ruby Lasers’ OR ‘SCx10 Carbon Dioxide Slab Laser’ OR ‘serial transplantation’ OR ‘Smartxide’ OR ‘SmartXide DOT’ OR ‘solid state carbon dioxide laser system’ OR ‘Solid State Laser’ OR ‘Solid State Laser’ OR ‘Solid State Lasers’ OR ‘specialties, surgical’ OR ‘Spectra’ OR ‘Subcutaneous Excision ‘ OR ‘surgery’ OR ‘surgical care’ OR ‘surgical correction’ OR ‘surgical diagnosis’ OR ‘surgical diagnostic techniques’ OR ‘surgical exposure’ OR ‘surgical grafting’ OR ‘surgical intervention’ OR ‘surgical management’ OR ‘surgical operation’ OR ‘surgical practice’ OR ‘Surgical Procedure’ OR ‘Surgical Procedures’ OR ‘surgical repair’ OR ‘surgical research’ OR ‘surgical restoration’ OR ‘surgical service’ OR ‘surgical special*’ OR ‘surgical therapy’ OR ‘surgical treatment’ OR ‘therapeutic uses’ OR ‘thrombocyte rich plasma’ OR ‘tissue and organ procurement’ OR ‘transplantation surgery’ OR ‘Transplantation*’ OR ‘transplants’ OR ‘UltraPulse Encore’ OR ‘UltraPulse Surgitouch’ OR ‘UM L30’ OR ‘Xenon Ion Laser’ OR ‘Xenon Ion Lasers’ OR ‘YAG Laser’ OR ‘YAG Lasers’ OR ‘YLF Laser’ OR ‘YLF Lasers’ OR ‘YSGG Laser’ OR ‘YSGG Lasers’ OR ‘Yttrium Aluminum Garnet Laser*’ OR ‘Yttrium Lithium Fluoride Laser*’ OR ‘Yttrium Scandium Gallium Garnet Laser*’ OR ‘Yttrium Lithium Fluoride Laser*’ OR ‘Yttrium Scandium Gallium Garnet Laser*’):ti,ab,kw |
| #12 | (#1 OR #2 OR #3) AND (#4 OR #5 OR #6 OR #7 OR #8 OR #9 OR #10 OR #11) |

## Search strategy of Web of Science.

| No. | Search items |
| --- | --- |
| #1 | "TS=((Acupulse) OR (Affirm neodymium YAG laser) OR (Alexandrite Laser*) OR (Argon Ion Laser*) OR (Autologous Fat Grafting) OR (Autologous Fat Transfer) OR (Carbon Dioxide Laser) OR (carbon dioxide laser device) OR (carbon dioxide laser systems) OR (Carbon Dioxide Lasers) OR (Chemotherap*) OR (CO2 Laser) OR (CO2 Lasers) OR (continuous wave carbon dioxide laser) OR (Copper Vapor Laser*) OR (CT3 Plus) OR (Diode Pumped Solid State Laser*) OR (dLase 300) OR (Drug Therap*) OR (drug treatment) OR (Ds-40u) OR (eCO2) OR (Er YAG Laser*) OR (Erbium YAG Laser*) OR (Erbium Doped Yttrium Aluminum Garnet Laser*) OR (Er-YAG Laser*) OR (Fractional Micro-needle Radiofrequency) OR (Fractional Micro-plasma Radio Frequency) OR (Fraxel Re:fine) OR (Gas Laser) OR (Gas Lasers) OR (general use solid state carbon dioxide laser) OR (general/multiple surgical solid-state/carbon dioxide laser system) OR (Ghost Surgery) OR (Gold Vapor Laser*) OR (graft surgery) OR (grafting surgery) OR (Helium Laser*) OR (Helium Neon Gas Lasers) OR (HeNe Laser*) OR (Ho YAG Laser*) OR (Holmium Laser*) OR (Holmium YAG Laser*) OR (Holmium Doped Yttrium Aluminum Garnet Lasers) OR (Holmium-YAG Laser*) OR (Ho-YAG Laser*) OR (JOULE ClearSense) OR (Juvia) OR (KTP Laser*) OR (LASER therapy) OR (laser treatment) OR (Laser Ablation) OR (Laser Knife*) OR (Laser Knive*) OR (Laser Photoablation of Tissue) OR (Laser Scalpel*) OR (Laser Surger*) OR (Laser Therap*) OR (Laser Tissue Ablation) OR (Laser Vaporization) OR (Lasertronics Paragon 50) OR (light amplification by stimulated emission of radiation therapy ) OR (medicament therapy) OR (medicament treatment) OR (medication) OR (medicinal therapy) OR (medicinal treatment) OR (Metal Vapor Laser*) OR (Mosaic CO2) OR (Nd YAG Laser*) OR (Nd:YAG laser) OR (Nd-YAG Laser*) OR (neodymium doped yttrium aluminum garnet laser) OR (neodymium YAG laser) OR (neodymium yttrium aluminium garnet laser) OR (neodymium yttrium aluminum garnet laser) OR (Neodymium Doped Yttrium Aluminum Garnet Laser*) OR (neodymium:YAG laser) OR (Neodymium-Doped Yttrium Aluminum Garnet Laser*) OR (Nitrogen Laser) OR (Nitrogen Lasers) OR (Non-ablative Fractional Erbium Glass Laser) OR (Nonablative Laser Treatment) OR (Nonablative Laser Treatments) OR (Opelaser-03 S) OR (operation) OR (operation care) OR (operative intervention) OR (Operative Procedure) OR (Operative Procedures) OR (operative repair) OR (operative restoration) OR (operative surgery) OR (Operative Surgical Procedure) OR (operative treatment) OR (Periolase MVP7) OR (pharmaceutical therapy) OR (pharmaceutical treatment) OR (pharmacological therapy) OR (pharmacological treatment) OR (Pharmacotherapies) OR (Pharmacotherapy) OR (pharmaco-therapy) OR (pharmacotreatment) OR (pharmaco-treatment) OR (PinPointe FootLaser) OR (Pixel Perfect) OR (Platelet Rich Plasma) OR (Potassium Titanyl Phosphate Laser) OR (Potassium Titanyl Phosphate Lasers) OR (Pulsed Laser Tissue Ablation) OR (Pulsemaster 600 IQ) OR (Q-Clear) OR (Radio Frequency Therapy) OR (Radio-Frequency Therapies) OR (radiofrequency therapy) OR (radiofrequency treatment) OR (radio-frequency treatment) OR (Radiofrequency Therapies) OR (Radiofrequency Therapy) OR (research surgery) OR (resection) OR (Ruby Laser) OR (Ruby Lasers) OR (SCx10 Carbon Dioxide Slab Laser) OR (serial transplantation) OR (Smartxide) OR (SmartXide DOT) OR (solid state carbon dioxide laser system) OR (Solid State Laser) OR (Solid-State Laser) OR (Solid-State Lasers) OR (specialties, surgical) OR (Spectra) OR (Subcutaneous Excision ) OR (surgery) OR (surgical care) OR (surgical correction) OR (surgical diagnosis) OR (surgical diagnostic techniques) OR (surgical exposure) OR (surgical grafting) OR (surgical intervention) OR (surgical management) OR (surgical operation) OR (surgical practice) OR (Surgical Procedure) OR (Surgical Procedures) OR (surgical repair) OR (surgical research) OR (surgical restoration) OR (surgical service) OR (surgical special*) OR (surgical therapy) OR (surgical treatment) OR (therapeutic uses) OR (thrombocyte rich plasma) OR (tissue and organ procurement) OR (transplantation surgery) OR (Transplantation*) OR (transplants) OR (UltraPulse Encore) OR (UltraPulse Surgitouch) OR (UM-L30) OR (Xenon Ion Laser) OR (Xenon Ion Lasers) OR (YAG Laser) OR (YAG Lasers) OR (YLF Laser) OR (YLF Lasers) OR (YSGG Laser) OR (YSGG Lasers) OR (Yttrium Aluminum Garnet Laser*) OR (Yttrium Lithium Fluoride Laser*) OR (Yttrium Scandium Gallium Garnet Laser*) OR (Yttrium-Lithium-Fluoride Laser*) OR (Yttrium-Scandium-Gallium Garnet Laser*)) " |
| #2 | "TS=((Acne) OR (acne keloid*) OR (Acne Vulgaris) OR (acneiform eruption) OR (acneiform eruptions) OR (acneiform skin eruption) OR (dermatitis papillaris capilliti*) OR (Folliculitis Keloidalis) OR (Folliculitis Keloidalis Nuchae) OR (keloid acne) OR (keloid folliculitis) OR (keloidal acne) OR (keloidal folliculitis) OR (Keloidal Acne*) OR (keloidalis nuchae acne) OR (Lichen Keloidalis Nuchae) OR (nuchal acne keloidalis) OR (nuchal keloid acne) OR (Nuchal Keloid Acnes) OR (Nuchal Keloid Acne) OR (pomade acne) OR (tropical acne)) " |
| #3 | #1 AND #2 |

# File S3: Characteristics of the included studies.

| **First author** | **Year** | **Country** | **Intervention** | **Samplesize** | **Sex(Male/Female)** | **Age** | **Skin type** | **Course** | **Follow-up** | **Outcome** |
| --- | --- | --- | --- | --- | --- | --- | --- | --- | --- | --- |
| Abdel Aal[28] | 2018 | Egypt | Lasers | 30 | 18/12 | 24.733±3.676 | III-V | NR | 6 months | GBS;Patient satisfaction |
|  |  |  | Lasers + PRP | 30 | 18/12 | 24.733±3.676 |  |  |  |  |
| Abdelwahab[29] | 2022 | Egypt | Filler injections + Subcision | 40 | 21/19 | 27.93±6.05 | II-IV | NR | 6 months | GBS; Patient satisfaction |
|  |  |  | Lasers + Subcision | 40 | 21/19 | 27.93±6.05 |  |  |  |  |
|  |  |  | Subcision | 40 | 21/19 | 27.93±6.05 |  |  |  |  |
| Abou Eitta[30] | 2019 | Egypt | Autologous fat grafting | 10 | 1/9 | 33.20±6.51 | NR | NR | 3 months | Patient satisfaction |
|  |  |  | Lasers | 10 | 1/9 | 33.20±6.51 |  |  |  |  |
| Afra[31] | 2018 | India | Microneedling | 36 | 13/23 | 23.4±2.9 | III-V | 3 months | 3/6 months | GBS |
|  |  |  | Drugs | 36 | 13/23 | 23.4±2.9 |  |  |  |  |
| Ahmed[32] | 2014 | Egypt | Lasers | 14 | 8/20 | 27.4±4.1 | II-V | NR | 6 months | GBS;Patient satisfaction; AE: PIH |
|  |  |  | Chemical peeling | 14 |  | 23.7±3.94 |  |  |  |  |
| Ali[33] | 2019 | Egypt | Microneedling | 20 | NR | NR | NR | NR | 3 months | GBS; AE: erythema |
|  |  |  | Chemical peeling | 20 |  |  |  |  |  |  |
|  |  |  | Microneedling + Chemical peeling | 20 |  |  |  |  |  |  |
| Amer[34] | 2021 | Egypt | Microneedling + PRP | 41 | 13/28 | 28±6.3 | II-V | NR | 1 months | GBS; Patient satisfaction |
|  |  |  | Microneedling + Filler injections | 41 | 13/28 | 28±6.3 |  |  |  |  |
| Anupama[35] | 2016 | India | Lasers + Subcision | 25 | NR | mean: 21 | Most IV | NR | 16 weeks | GBS;Patient satisfaction |
|  |  |  | Lasers | 25 |  |  |  |  |  |  |
| Arsiwala[36] | 2020 | India | Lasers + PRP | 17 | 21/12 | 24.36±4.37 | III-V | 3 months | 12weeks | GBS |
|  |  |  | Lasers | 16 |  |  |  |  |  |  |
| Bano[37] | 2023 | India | Microneedling | 30 | 11/19 | 26.90±4.90 | NR | NR | 17 weeks | GBS; AE: PIH, infection |
|  |  |  | Microneedling + Filler injections | 30 | 13/17 | 24.43±5.39 |  |  |  |  |
| Behrangi[38] | 2021 | Iran | Microneedling | 30 | 7/23 | 32.73±7.88 | NR | NR | 3 months | GBS |
|  |  |  | Microneedling + PRP | 22 | 1/22 | 32.95±8.20 |  |  |  |  |
|  |  |  | Lasers | 26 | 7/19 | 29.64±6.27 |  |  |  |  |
| Bhargava[39] | 2019 | India | Microneedling + Subcision | 15 | 6/9 | mean: 27.1 | III-V | NR | 3 months | GBS |
|  |  |  | Microneedling + PRP + Subcision | 15 | 5/10 | mean: 28.2 |  |  |  |  |
| Brar[40] | 2024 | India | Lasers | 25 | 16/9 | 20-45 | NR | NR | 3 months | GBS; Patient satisfaction |
|  |  |  | Microneedling | 25 | 16/9 |  |  |  |  |  |
| Cachafeiro[41] | 2016 | Brazil | Microneedling | 20 | 10/10 | mean: 27.35 | II-IV | NR | 6 months | GBS; AE: PIH |
|  |  |  | Lasers | 22 | 11/11 | mean: 25.41 |  |  |  |  |
| Chae[42] | 2014 | Korea | Lasers | 20 | 7/13 | 25.5±3.76 | III-V | NR | 20 weeks | ECCA; VAS; AE: erythema, PIH |
|  |  |  | Microneedling | 20 | 16/4 | 28.3±5.39 |  |  |  |  |
| Chary[79] | 2022 | Pakistan | Microneedling | 70 | NR | NR | >III | 12 months | 6 months | GBS; Patient satisfaction |
|  |  |  | Lasers | 70 |  |  |  |  |  |  |
| Chen[43] | 2024 | China | Lasers + Filler injections | 12 | 8/4 | 25.58±4.52 | I-IV | NR | 2 months | ECCA; AE: erythema |
|  |  |  | Lasers + Drugs | 12 | 8/4 | 25.58±4.52 |  |  |  |  |
| Dastgheib[44] | 2024 | Iran | Subcision | 12 | 3/9 | mean: 29.75 | II-IV | NR | 3  months | SQGS; AE: erythema, petechiae, PIH |
|  |  |  | Filler injections + Subcision | 12 | 3/9 |  |  |  |  |  |
| Deepika[45] | 2024 | India | Microneedling + Chemical peeling | 30 | 18/12 | 27.1±5.31 | III-VI | NR | 24 weeks | ECCA; Patient satisfaction |
|  |  |  | Microneedling + PRP | 30 | 20/10 | 25.1±4.84 |  |  |  |  |
| Diab[46] | 2023 | Egypt | PRP | 15 | 2/13 | 24.53±4.81 | most IV | NR | 1 months | GBS; Patient satisfaction |
|  |  |  | Microneedling + PRP | 15 | 2/13 | 24.53±4.81 |  |  |  |  |
| Ebrahim[47] | 2021 | Egypt | Subcision | 34 | 18/22 | 26.65±6.77 | NR | NR | 6 months | GBS; Patient satisfaction; AE: erythema, edema |
|  |  |  | Filler injections + Subcision | 34 |  |  |  |  |  |  |
| El-Domyati[48] | 2018 | Japan | Microneedling | 24 | 6/18 | 27.33±4.05 | III-IV | 2months | 3 months | Patient satisfaction |
|  |  |  | Microneedling + PRP |  |  |  |  |  |  |  |
|  |  |  | Microneedling + Chemical peeling |  |  |  |  |  |  |  |
| Elfar[50] | 2020 | Egypt | Drugs | 60 | 24/36 | 27.10±5.07 | II-V | NR | 3 months | GBS |
|  |  |  | Microneedling |  |  |  |  |  |  |  |
|  |  |  | Microneedling + Drugs |  |  |  |  |  |  |  |
| El-Taieb[49] | 2019 | Egypt | PRP | 75 | 27/48 | 26.7±5.1 | III-IV | PRP: 2 weeks laser: 4 weeks | NR | GBS; Patient satisfaction; AE: erythema, PIH |
|  |  |  | Lasers |  |  |  |  |  |  |  |
|  |  |  | Lasers + PRP |  |  |  |  |  |  |  |
| Emam[51] | 2021 | Egypt | Lasers | 21 | 9/12 | 27.29±6.10 | II-IV | 4 months | 3 months | GBS;VAS; Patient satisfaction |
|  |  |  | Microneedling | 21 | 9/12 | 27.29±6.10 |  |  |  |  |
| Faghihi[53] | 2015 | Iran | Microneedling + Lasers | 42 | 23/19 | 23.4±2.63 | III-IV | 5 weeks | 4 months | GBS; VAS |
|  |  |  | Lasers |  |  |  |  |  |  |  |
| Faghihi[52] | 2016 | Iran | Lasers + PRP | 16 | 4/12 | mean: 36.8 | II-IV | NR | NR | GBS; Patient satisfaction; VAS |
|  |  |  | Lasers |  |  |  |  |  |  |  |
| Faghihi[54] | 2017 | Iran | Microneedling + Subcision | 25 | 9/16 | 30.08 ± 4.94 | II-IV | 3 months | 3 months | GBS; VAS |
|  |  |  | Microneedling | 25 | 9/16 | 30.08 ± 4.94 |  |  |  |  |
| Guo[56] | 2022 | China | Lasers + Drugs | 52 | 28/24 | 23:46±3.01 | III-IV | 4 months | 3 months | Patient satisfaction; AE: erythema, edema |
|  |  |  | Lasers | 57 | 28/29 | 23:09±3:06 |  |  |  |  |
| Guo [55] | 2023 | China | Lasers + PRP | 39 | 23/16 | 24.7 ± 5.8 | NR | NR | 24 months | Patient satisfaction; AE: edema |
|  |  |  | Lasers | 42 | 25/17 | 25.0 ± 5.0 |  |  |  |  |
| Gupta[57] | 2021 | India | Microneedling | 36 | 17/19 | 23.7±3.2 | NR | 4 months | 6 months | ECCA |
|  |  |  | PRP | 36 | 17/19 | 23.7±3.2 |  |  |  |  |
| Han[58] | 2021 | China | Lasers + Drugs | 9 | 5/4 | 31.56±10.48 | NR | 3 months | 3 months | ECCA |
|  |  |  | Lasers | 9 | 3/6 | 26.44±10.32 |  |  |  |  |
| Hawwas[59] | 2023 | Egypt | Lasers | 30 | 11/19 | 25.63±3.76 | NR | 5 weeks | 8 weeks | Patient satisfaction; AE: erythema, edema, PIH |
|  |  |  | Lasers + Chemical peeling |  |  |  |  |  |  |  |
| Hendel[60] | 2023 | Denmark | Lasers | 15 | 2/13 | NR | II-III | NR | 3 months | VAS; Patient satisfaction |
|  |  |  | Microneedling |  |  |  |  |  |  |  |
| Huang[61] | 2023 | China | Lasers + Chemical peeling | 30 | 11/19 | 24.87±3.63 | II-IV | NR | 18 weeks | ECCA; VAS; Patient satisfaction |
|  |  |  | Lasers |  |  |  |  |  |  |  |
| Ismail[62] | 2022 | Egypt | Microneedling + PRP | 15 | 6/9 | 25.60±4.84 | III-V | 3 months | 3 months | GBS; ECCA; AE: erythema, edema |
|  |  |  | Microneedling | 15 | 6/9 | 25.60±4.84 |  |  |  |  |
|  |  |  | Microneedling + PRP | 15 | 5/10 | 23.53±5.24 |  |  |  |  |
|  |  |  | PRP | 15 | 5/10 | 23.53±5.24 |  |  |  |  |
| Jangir[63] | 2023 | India | Lasers | 25 | 5/20 | 26.6±5.72 | NR | NR | 3 months | GBS; AE: erythema, edema, PIH |
|  |  |  | Chemical peeling | 25 | 14/11 | 27.68±6.50 |  |  |  |  |
| Kamel[64] | 2021 | Egypt | Subcision + PRP | 20 | 5/15 | 30.05±9.79 | III-IV | 3 months | 1 months | GBS; AE: erythema, edema, PIH |
|  |  |  | Subcision + PRP + Chemical peeling | 20 | 5/15 | 30.05±9.79 |  |  |  |  |
| Kaur[65] | 2020 | India | Lasers | 21 | NR | NR | NR | NR | 2 months | GBS |
|  |  |  | Subcision |  |  |  |  |  |  |  |
| Kwon[66] | 2017 | Korea | Microneedling + Lasers | 28 | 15/13 | 21–38 | III-IV | 4 months | 2 months | ECCA |
|  |  |  | Lasers | 28 | 15/13 |  |  |  |  |  |
| Liao[67] | 2023 | China | Lasers + Filler injections | 50 | NR | 20.6±2.3 | NR | NR | 6 months | ECCA; AE: edema, infection |
|  |  |  | Lasers | 50 |  | 21.1±2.6 |  |  |  |  |
| Medhat[68] | 2024 | Egypt | Drugs | 26 | 12/14 | 28.12±5.4 | NR | 3months | 3 months | GBS; Patient satisfaction |
|  |  |  | Lasers |  |  |  |  |  |  |  |
| Memon[69] | 2022 | Pakistan | Microneedling | 40 | 18/22 | 31±6.1 | NR | 3 months | NR | Patient satisfaction |
|  |  |  | Chemical peeling | 40 | 17/23 | 27.3±7.8 |  |  |  |  |
|  |  |  | Microneedling + Chemical peeling | 40 | 16/24 | 26.1±7.1 |  |  |  |  |
| Mukhtar[70] | 2023 | Pakistan | Lasers | 188 | 143/45 | 28.15±5.43 | II-III | NR | 5 months | GBS; Patient satisfaction |
|  |  |  | Microneedling |  |  |  |  |  |  |  |
| Mumtaz[71] | 2021 | Pakistan | PRP | 46 | 27/19 | 27.72±8.05 | NR | 3 months | 6 months | GBS |
|  |  |  | Chemical peeling | 46 | 22/24 | 27.72±8.05 |  |  |  |  |
| Nandini[72] | 2021 | India | Microneedling + PRP | 30 | 18/12 | 25 (19-35) | II-IV | 4 months | 6 months | GBS; Patient satisfaction |
|  |  |  | Microneedling | 30 | 18/12 | 25 (19-35) |  |  |  |  |
| Osman[73] | 2016 | Egypt | Lasers | 30 | 20/10 | 27±3.75 | III-V | 5 months | 3 months | SQGS |
|  |  |  | Microneedling | 30 | 20/10 | 27±3.75 |  |  |  |  |
| Pooja[74] | 2020 | India | Lasers | 60 | 42/18 | mean: 25.4 | NR | 4months | NR | GBS; AE: erythema, edema, PIH |
|  |  |  | Microneedling |  |  |  |  |  |  |  |
|  |  |  | PRP |  |  |  |  |  |  |  |
| Porwal[75] | 2018 | India | Microneedling + PRP | 55 | 24/31 | 26.01±3.6 | III-V | NR | NR | GBS; VAS; AE: erythema, edema, PIH |
|  |  |  | Microneedling |  |  |  |  |  |  |  |
| Prathyusha[76] | 2024 | India | Microneedling + PRP | 30 | NR | NR | NR | NR | 4 months | AE: erythema, PIH |
|  |  |  | Microneedling |  |  |  |  |  |  |  |
| Pratiwi[77] | 2020 | Indonesia | Lasers + Drugs | 9 | 8/10 | 33.7±6.02 | NR | 3 months | 3 months | GBS |
|  |  |  | Lasers | 9 | 8/10 | 30.0±7.95 |  |  |  |  |
| Priya[78] | 2023 | India | Lasers + PRP | 32 | 18/14 | 18-40 | NR | NR | 6 weeks | GBS; VAS; Patient satisfaction |
|  |  |  | Lasers | 32 |  |  |  |  |  |  |
| Rana[80] | 2017 | India | Microneedling | 30 | NR | 23.30±4.41 | IV-V | 3 months | 3 months | ECCA; VAS; AE: erythema |
|  |  |  | Chemical peeling | 30 |  | 23.30±4.41 |  |  |  |  |
| Saadawi[81] | 2018 | Egypt | Microneedling | 30 | 10/20 | 29.17±7.08 | II-IV | 2 weeks | 1 month | GBS; Patient satisfaction; AE: erythema |
|  |  |  | Chemical peeling |  |  |  |  |  |  |  |
|  |  |  | Microneedling + Chemical peeling |  |  |  |  |  |  |  |
| Sage[82] | 2011 | USA | Filler injections | 10 | NR | 49.58±10.34 | II-V | NR | 6 months | Patient satisfaction |
|  |  |  | Subcision | 10 |  |  |  |  |  |  |
| Saleem[83] | 2022 | Pakistan | Lasers + Filler injections | 300 | 90/210 | 23.2±4.7 | NR | 3 months | 1 months | GBS |
|  |  |  | Lasers | 300 | 90/210 | 23.2±4.7 |  |  |  |  |
| Sharad[84] | 2011 | India | Microneedling | 30 | 8/22 | 20-40 | III-V | 6 weeks | 3 months | ECCA |
|  |  |  | Microneedling + Chemical peeling |  |  |  |  |  |  |  |
| Sharma[86] | 2020 | India | Microneedling + PRP | 40 | 13/27 | 25.17±7.16 | II-IV | 4 months | 6 months | GBS; AE: erythema, PIH |
|  |  |  | Microneedling | 40 | 13/27 | 25.17±7.16 |  |  |  |  |
| Sharma[85] | 2021 | India | Lasers + PRP | 30 | 1.3/1 | 26.93±4.77 | III-IV | 4 months | 2 months | GBS; AE: erythema, edema, PIH |
|  |  |  | Lasers | 30 | 1.3/1 | 26.93±4.77 |  |  |  |  |
| Shetty[87] | 2021 | India | Autologous fat grafting | 12 | 7/17 | 33.58±7.42 | NR | 3 months | 3 months | GBS; Patient satisfaction; AE: erythema,edema |
|  |  |  | Subcision + PRP | 12 | 7/17 | 35.18±6.32 |  |  |  |  |
| Taleb[88] | 2024 | Israel | Lasers | 16 | 5/11 | 20.4±5.4 | NR | NR | 6 months | GBS; VAS |
|  |  |  | Drugs |  |  |  |  |  |  |  |
| Wang[17] | 2021 | China | Lasers + PRP | 150 | 64/86 | 20.20±2.11 | NR | 3 months | NR | ECCA; AE: erythema, edema, PIH |
|  |  |  | Lasers | 150 | 66/84 | 20.14±2.07 |  |  |  |  |
| Yang[89] | 2016 | China | Lasers | 26 | 14/12 | 18-65 | NR | NR | 164 days | Patient satisfaction |
|  |  |  | Drugs | 26 |  |  |  |  |  |  |
| Yang[90] | 2019 | China | Microneedling + Lasers | 20 | 8/12 | NR | III-IV | 9 months | 3 months | ECCA; VAS; AE |
|  |  |  | Lasers | 20 | 8/12 |  |  |  |  |  |
| Yuan[91] | 2023 | China | Lasers + Drugs | 60 | 26/34 | 21.2±3.5 | NR | NR | NR | ECCA; AE: erythema |
|  |  |  | Lasers | 60 | 24/36 | 21.7±3.4 |  |  |  |  |
| Zayed[92] | 2021 | Egypt | Microneedling | 20 | 9/11 | 28.55±8.77 | III-IV | 6 months | 1 months | ECCA; AE: erythema, PIH |
|  |  |  | Chemical peeling | 20 | 9/11 | 28.55±8.77 |  |  |  |  |
| Zhang[94] | 2013 | china | Microneedling | 33 | 19/14 | 26.4±3.7 | NR | 3months | 6 months | ECCA; Patient satisfaction; AE: PIH |
|  |  |  | Lasers |  |  |  |  |  |  |  |
| Zhang[93] | 2022 | China | Lasers + Chemical peeling | 20 | 16/4 | 23.65±3.18 | III-IV | 3 months | 3 months | ECCA; VAS; AE: erythema, edema, PIH |
|  |  |  | Lasers | 20 | 16/4 | 23.65±3.18 |  |  |  |  |

Abbreviations: NR:Non Reporting; GBS:Goodman and Baron Scale; ECCA:Echelle d'evaluation clinique des cicatrices d'acne; VAS:visual analogue scale; AE:Adverse reaction; PIH:postinflammatory hyperpigmentation

# Target Audience Description

**Dermatologists and Skin Care Specialists:** This study provides dermatologists with an evidence-based comparison of various treatments for acne scars, helping them to choose the most effective therapies for their patients. The findings can guide clinical practice towards more personalized and efficient treatment strategies.

**Plastic Surgeons and Aesthetic Practitioners:** Professionals specializing in aesthetic medicine will find this analysis useful for understanding the comparative effectiveness of different procedures available for treating acne scars. It supports informed decision-making in cosmetic and reconstructive surgery settings.

**Researchers and Academics:** Investigators focused on dermatology and related fields can use this comprehensive review as a resource for identifying current best practices and emerging trends in acne scar treatment. It highlights potential areas for further research and innovation.

**Clinical Guideline Developers:** Organizations responsible for developing clinical guidelines can leverage the synthesized evidence from this study to update or create new recommendations that reflect the latest understanding of effective treatments for acne scars.

**Patients and Patient Advocates:** Individuals affected by acne scars and those advocating on their behalf can gain valuable insights into the range of treatment options available. This empowers patients to have more informed discussions with healthcare providers about suitable treatment plans.

**Healthcare Policy Makers:** Policymakers can use the results of this meta-analysis to inform health policy decisions, ensuring that public health resources are allocated efficiently towards proven and cost-effective treatments for acne scars.

**Pharmaceutical and Biotechnology Companies:** Companies developing products for skin care and dermatological treatments can utilize the findings to focus their R&D efforts on the most promising therapeutic approaches. It also aids in marketing strategies by highlighting the benefits of their products compared to alternatives.
